# c-Myc-PANK3-EMT axis regulates the structure and function of intestinal barrier in ulcerative colitis

**DOI:** 10.1016/j.jare.2025.12.007

**Published:** 2025-12-08

**Authors:** Shize Zhang, Yuang Chen, Nan Aa, Chen Xu, Tao Xie, Yi Wang, Tian Cheng, Mengqin Wang, Hong Yu, Xinqiang Ji, Song Zhao, Yaohui Wang, Jun Xiao, Yuan Xie, Guangji Wang, Jiye Aa

**Affiliations:** a Key Laboratory of Drug Metabolism and Pharmacokinetics, State Key Laboratory of Natural Medicines, China Pharmaceutical University, Nanjing, PR China; b Department of Gastroenterology, The First Affiliated Hospital with Nanjing Medical University, Nanjing, PR China; c Digestive Endoscopy Center, Jiangsu Province Hospital of Chinese Medicine, Affiliated Hospital of Nanjing University of Chinese Medicine, Nanjing, PR China; d Department of Gastroenterology, Jiangsu Province Hospital of Chinese Medicine, Affiliated Hospital of Nanjing University of Chinese Medicine, Nanjing, PR China; e Department of Pathology, Jiangsu Province Hospital of Chinese Medicine, Affiliated Hospital of Nanjing University of Chinese Medicine, Nanjing, PR China

**Keywords:** Ulcerative colitis, Intestinal barrier, PANK3, c-Myc, EMT, Folic acid

## Abstract

**Introduction:**

The repair of structural damage of the intestinal barrier is critical to rescue ulcerative colitis (UC), but there lacks effective targets currently.

**Objectives:**

This study aims to explore mechanisms and identify potential targets for repairing the damaged intestinal barrier involved in UC.

**Methods:**

Nontargeted metabolomic and transcriptomic analyses were utilized to characterize the metabolic features of DSS-induced colitis and identify potential therapeutic targets. Pantothenate kinase 3 (PANK3) was pharmacologically and genetically modulated to elucidate its mechanistic role. Chromatin immunoprecipitation and dual-luciferase reporter assays were employed to identify upstream regulators of PANK3, and PANK3 agonists were screened by high-throughput docking and further confirmed by *in vitro* and *in vivo* experiments.

**Results:**

Metabolomic data indicated that perturbed metabolism involves pantothenate kinase. Clinical database, qPCR, WB, and immunohistochemical staining revealed a significant decrease of PANK3 in both patients and murine models of colitis. Pharmacological activation or overexpression of PANK3 restored the integrity of the intestinal barrier in UC mice, whereas PANK3 knockdown or inhibition exacerbated barrier dysfunction. PANK3 is negatively regulated by upstream c-Myc, and affects downstream, epithelial‒mesenchymal transition (EMT). In general, elevated c-Myc suppressed PANK3 transcription, promoted the EMT process and induced barrier disruption. The natural vitamin folic acid was identified as an agonist of PANK3, which could markedly improve the intestinal barrier in DSS-induced colitis.

**Conclusion:**

The c-Myc-PANK3-EMT axis plays a critical role in preserving the structure and function of intestinal barrier. PANK3 is a potential target and folic acid is a candidate agonist of PANK3 to repair intestinal barrier in UC.

## Highlights

• Intestinal epithelial PANK3 is down-regulated in ulcerative colitis.
• Upregulated PANK3 reconstructs the impaired intestinal epithelial barrier *in vitro* and *in vivo*.
• c-Myc represses PANK3 to drive EMT-mediated intestinal barrier damage.
• The naturally derived vitamin folic acid activates PANK3 to restore the intestinal barrier and alleviate colitis.

## Graphical abstract

The c-Myc-PANK3 axis drives intestinal barrier dysfunction in ulcerative colitis by regulating downstream EMT-mediated epithelial damage. Thus, PANK3 represents a novel therapeutic target for UC.

**Figure:**
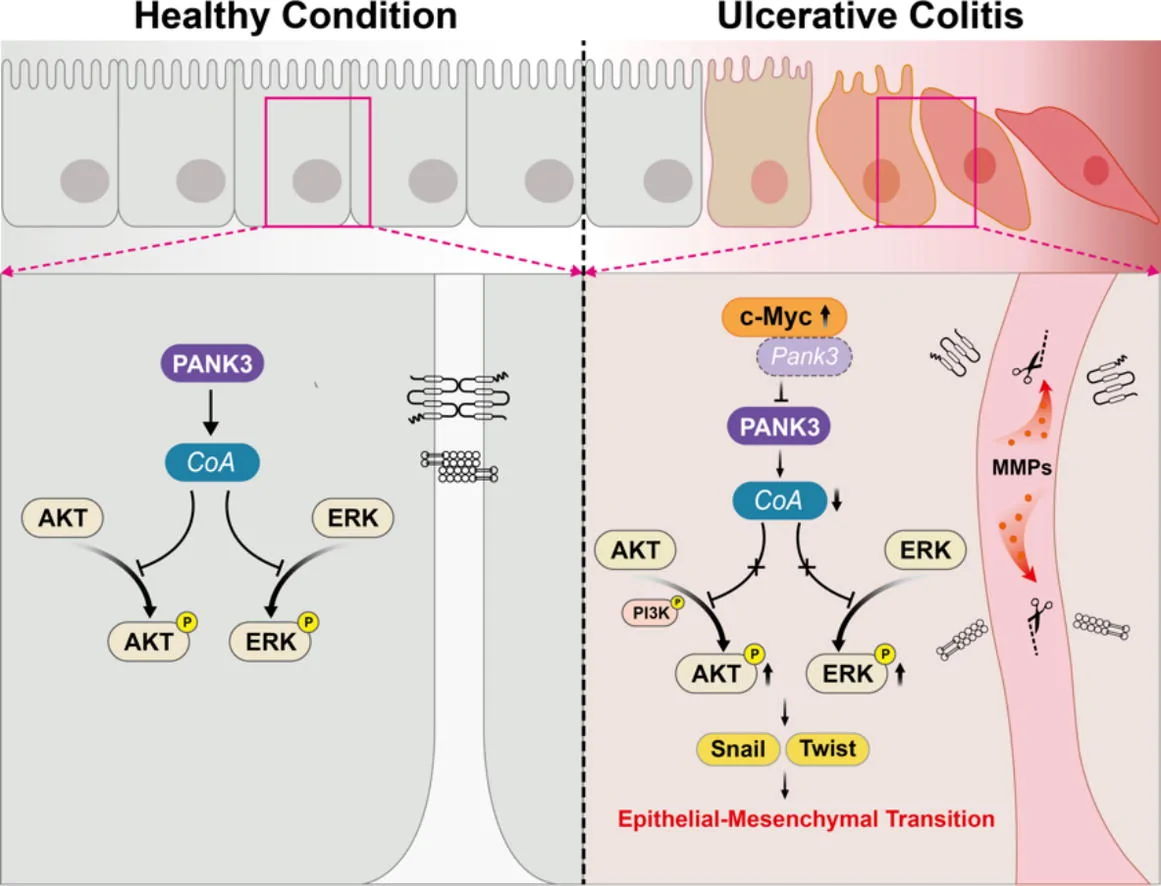

## Introduction

Inflammatory bowel disease (IBD), represented by Crohn’s disease (CD) and ulcerative colitis (UC), is a chronic, relapsing idiopathic gastrointestinal inflammatory disease that results in severe disability [1]. The number of IBD patients has grown exponentially in recent decades, resulting in a substantial health care burden globally [2]. Current therapeutic strategies for IBD include traditional agents such as aminosalicylates, corticosteroids and immunosuppressants, as well as targeted therapies such as biologic antibodies, Janus kinase (JAK) inhibitors, and sphingosine-1-phosphate receptor (S1PR) modulators [3]. However, these treatments often suffer from suboptimal efficacy, high relapse rates, and unavoidable adverse effects [4,5]. The primary reasons for the present clinical dilemma in IBD management are that the key pathogenic mechanisms have not been elucidated and that there is a lack of therapeutic targets. The pathogenesis of IBD involves a complex interplay of immune system dysfunction, intestinal microbe disorders, intestinal barrier disruption and genetic susceptibility [6,7]. Among these factors, the immune-mediated inflammatory response is considered the fundamental mechanism of IBD. However, the efficacy of both antibodies against proinflammatory cytokines such as TNF-α, IL-12/23, and IL-17 and small-molecule drugs that inhibit overactivated mucosal immunity is limited in a specific subset of patients [8,9]. These findings underscore the need for further research to elucidate the intricate IBD underlying mechanisms and to identify novel therapeutic targets.

The intestinal barrier, which is predominantly composed of a mucus layer and an intestinal epithelial barrier, functions as a pivotal defensive mechanism separating the internal milieu from the external environment [10,11]. The barrier is essential for intestinal homeostasis and plays a central role in overall gut health. Although disruption of the intestinal barrier is associated with IBD, the specific relationship between the two, such as whether intestinal barrier damage is a cause or a consequence of IBD, remains unknown. Nevertheless, recent clinical studies have indicated that increased intestinal permeability occurs prior to the progression of disease, and that restoration of barrier integrity is a strong predictor of treatment outcomes in patients with IBD [[12], [13], [14]]. These findings highlight the pivotal role of the intestinal barrier in the development and management of IBD, emphasizing its potential as a therapeutic target. Specific genes associated with gut barrier function are considered prospective therapeutic targets for future IBD treatment [[15], [16], [17]]. Moreover, as the cornerstone of the epithelial barrier, intestinal epithelial cells (IECs) are highly susceptible to endogenous metabolism due to their high turnover rate [18,19]. Notably, the integrity of the intestinal barrier is also compromised in certain extraintestinal metabolic disorders, such as obesity and diabetes [20,21]. Conversely, various functional metabolites have demonstrated potential for modulating the intestinal barrier [22,23]. Although the relationship between metabolic abnormalities and barrier damage or repair has not yet been fully elucidated, this study offers a novel perspective for identifying effective targets for restoring barrier function in the treatment of IBD.

Therefore, based on the aforementioned background, this study employs a systems biology approach to comprehensively analyze metabolic abnormalities and their molecular mechanisms underlying intestinal barrier dysfunction. By integrating analyses of the intestinal microenvironment and specific substance changes with transcriptomic profiling, this research aims to uncover novel mechanisms and identify potential therapeutic targets for the treatment of UC.

## Material and methods

### Human samples collection

Colon biopsy samples were provided from Jiangsu Province Hospital and Jiangsu Province Hospital of Chinese Medicine, comprising a total of 10 participants (4 healthy controls, 3 ulcerative colitis patients, and 3 Crohn’s disease patients). Written informed consent was obtained from all clinic participants prior to sample collection. This study was approved by Institutional Review Board of Jiangsu Province Hospital (2023-SR-980) and Jiangsu Province Hospital of Chinese Medicine (2022NL-069–02), respectively.

### Cell culture and stimulation conditions

HT29, Caco-2 and HEK-293T were purchased from Procell (Wuhan, China). Cells were cultured in Dulbecco’ s modified Eagle’s medium (DMEM) supplemented with 10 % Fetal Bovine Serum (FBS) and 1 % penicillin/streptomycin excerpt for Caco-2 which was maintained in DMEM with 20 % FBS. NCM-460 was purchased from iCell (Shanghai, China) and cultured in RPMI-1640 with 10 % FBS and 1 % penicillin/streptomycin. All cell lines were authenticated by STR profiling and tested for mycoplasma contamination. The cells were treated with 30 ng/mL TNF-α (PeproTech, USA) and IFN-γ (PeproTech) in the presence or absence of PZ-2891 (2 µM, MCE, USA), Hopantenate (250 µM, Macklin, China), CoA (250 µM, MCE) and folic acid (1 µM, MCE) respectively for 12 h. For c-Myc inhibition, HT29 cells were treated with 10058-F4 (10 µM and 20 µM, MCE) for 12 h.

### Mice and drug administration

6-weeks-old male C57BL/6J mice were purchased from Charles River (Zhejiang, China). 8-weeks-old male IL-10 deficient mice (n = 5) were obtained from Cyagen Biosciences (Suzhou, China). Mice were housed under SPF conditions (20 ± 2°C, 40–60 % humidity, 12-hour/12-hour light/dark cycle) and approved by the Ethical Committee for Experimental Animal Care of China Pharmaceutical University (2023–01-012). Mice were stratified into experimental cohorts through body weight-matching to ensure intergroup homogeneity. All following compounds were administrated by oral gavage. The specific formulations and dosages were as follows: PZ-2891 was dispersed in 30 % captisol (Aladdin, China) at a concentration of 3 mg/mL and administered at 30 mg/kg; 10058-F4 and folic acid (MCE) were homogeneously suspended in 0.5 % sodium carboxymethyl cellulose (CMC-Na) at concentrations of 5 mg/mL and 3 mg/mL, respectively, and administered at 50 mg/kg and 30 mg/kg; Hopantenate was dissolved in saline at a concentration of 20 mg/mL and administered at 200 mg/kg. Vehicle control groups received the corresponding solvents (30 % captisol, 0.5 % CMC-Na, or saline) at the same administration volume.

### Dss-induced experimental colitis

Acute experimental colitis in mice (n = 8, per group) was induced by giving 2–3 % DSS (w/v, 36–50 KD, MP biomedicals, USA) in drinking water for 7 days. After 7 days of induction, mice were anesthetized with isoflurane and colon samples were collected for further analysis.

### Overexpression constructs and shRNA knockdown

Overexpression plasmids of human PANK3 and its control, human c-Myc and its control were purchased from VectorBuilder (Guangzhou, China). shRNA knockdown of human PANK3 and its control were obtained from Genepharma (Shanghai, China). Plasmids transfection was performed using Lipofectamine^TM^ 3000 (Invitrogen, USA) according to the manufacturer’s instruction. Target sequences are as follows: 5′-3′: ACCTTGAACTGAAAGATTTAAT. 3′-5′: TTTGGAACTTGACTTTCTAAATTA.

### Virus injection

The overexpression adeno-associated virus AAV9-CMV-PANK3-P2A-GFP (AAV-*Pank3*-OE, 4.64 × 10^13^ vg/mL) and its control virus AAV9-CMV-GFP (AAV-GFP, 6.50 × 10^13^ vg/mL) were constructed by WZ Biosciences (Jinan, China). Similarly, the knockdown virus AAV9-CMV-4in1shRNA-PANK3-GFP (AAV-sh*Pank3*, 2.27 × 10^13^ vg/mL) and its control virus AAV9-CMV-Scramble-GFP (AAV-shNC, 5.53 × 10^13^ vg/mL) were also obtained from the same vendor. All viral stocks were diluted in sterile ice-cold PBS to a working concentration of 1.0 × 10^12^ vg/mL and a total volume of 100 µL of the respective virus (equivalent to 1.0 × 10^11^ vector genomes per mouse) was administered via enema. Transgene expression was allowed to stabilize for four weeks before the subsequent DSS-induced modeling.

### Immunoblotting

Total cellular and tissue proteins were extracted with RIPA lysates (Beyotime, China); Nuclear and cytoplasmic protein separation was performed using a Nuclear and Cytoplasmic Protein Extraction Kit (Beyotime). Protein samples were then separated by SDS-PAGE, transferred to PVDF membranes, and blocked in 5 % skimmed milk for 2 h at RT. After incubated overnight at 4 °C with the appropriate primary antibodies, the membranes were washed and incubated with corresponding HRP-conjugated secondary antibodies for 2 h at RT. All blots were visualized by gel-imaging system (Bio-Rad, USA). Antibodies used in this study are listed in online supplemental table S1.

### Immunohistochemistry and immunofluorescence staining

For IHC, tissue sections were fixed, paraffin-embedded, and cut into thin slices. After deparaffinization, antigen retrieval, and blocking, slides were incubated with primary antibodies overnight at 4 °C, followed by secondary antibodies conjugated with horseradish peroxidase (HRP). Staining was visualized using diaminobenzidine (DAB), and nuclei were counterstained with hematoxylin. Images were taken by Nanozoomer S60 (HAMAMATSU, Japan). For IF, cells or tissue sections were fixed with paraformaldehyde, permeabilized with 0.3 % Triton X-100, and blocked with 5 % bovine serum albumin (BSA). Primary antibodies were applied overnight at 4 °C, followed by fluorophore-conjugated secondary antibodies. Nuclei were stained with DAPI, and images were captured using fluorescence microscope FV3000 (Olympus, Japan). Antibodies used in this study are listed in online supplemental table S1.

### Quantitive RT-PCR and bulk RNA sequencing

Total RNA of cells or tissues were extracted using TRIzol (Vazyme, China), quantified, and then reverse transcribed to cDNA using Hiscript Reverse Transcriptase Kit (Vazyme). Quantitive RT-PCR was performed using One Step qRT-PCR Probe Master Mix (Vazyme) on a CFX96 Real Time PCR-Cycler (Bio-Rad). The relative mRNA expression was determined by ΔΔCt method and normalized to GAPDH. The primer sequences are listed in online supplemental table S2. For RNA sequencing, colon tissues were rapidly frozen in liquid nitrogen and then used to extract total RNA. After purification and quality assessment, sequencing analysis was performed on an Illumina NovaSeq 6000 platform to generate high-quality paired-end reads by Gene Devono (Guangzhou, China). Differential expression analysis was conducted using DESeq2 and visualization of results was generated using online platform Omicsmart.

### Chromatin immunoprecipitation and qPCR

Pierce^TM^ Magnetic ChIP Kit (CatLog No. 26157) was purchased from Thermofisher (USA) and carried out according to manufacturer’s instruction. Briefly, HT29 cells were cross-linked with 1 % formaldehyde for 10 min at RT to preserve protein-DNA interactions, followed by quenching with 125 mM glycine. Chromatin was sheared to 150–900 bp fragments using a SANYO Soniprep 150 at 10 μm amplitude, with four cycles of 20-second pulses interspersed with 20-second intervals on ice. The sample tube was kept in an ice-water bath throughout to maintain 4 °C. The lysate was incubated with a specific antibody to c-Myc (abcam). To ensure the specificity of the immunoprecipitation, parallel samples were processed using a species-matched normal IgG as a negative control and an antibody against RNA Polymerase II as a positive control. Subsequently, immune complexes from all samples were captured using protein A/G beads, washed extensively with the kit's IP wash buffer, and eluted. Cross-links were reversed by heating at 65 °C, and DNA was purified using DNA clean-up columns. q-PCR analysis of enriched DNA fragments was performed for different binding sites between c-Myc and *pank3* promoter. The ChIP primer sequences are listed in online supplemental table S2.

### Dual-Luciferase reporter assay

Firefly luciferase reporter plasmid containing full-length *pank3* promoter (GPL4-promotor-luci), GPL4-luci, GPL4-basic, and GPL4-RL plasmids were purchased from Genepharma (Shanghai, China). In brief, HEK-293T cells were seeded in 12-well plates and co-transfected with GPL4-promotor-luci or its control GPL4-basic, c-Myc overexpression plasmid or its control, and a Renilla luciferase control plasmid using Lipofectamine^TM^ 3000. After 48 h of incubation, luminescence of both luciferases was determined using Dual Luciferase Reporter Gene Assay Kit (Yeasen, China). The relative luciferase activity was calculated as the ratio of Firefly luciferase activity to Renilla luciferase activity.

### Cellular thermal shift assay

CETSA was conducted based on previous studies [24]. In brief, HEK-293T cells were inoculated into 60 mm dishes and transfected with the Flag-tagged PANK3 expression plasmid. After incubation for 48 h, DMSO and 20 µM folic acid were administered for 12 h. Cells were suspended in ice PBS and evenly divided into 8 equal portions for heating at different temperatures (55, 57, 59, 61, 63, 65, 67, 69 °C) for 5 min. Cell suspensions were repeatedly freeze-thawed three times for protein extraction, which was then used for immunoblotting analysis.

### Colon coenzyme A determination

Colon tissue (20 mg) was homogenized and extracted in 200 µL of cooled ddH2O, followed by the addition of 800 µL of cooled methanol (containing 20 ng/mL tenofovir). After centrifugation at 20,000g × 10 min, 600 µL of the supernatant was nitrogen-blown and evaporated, followed by the addition of 100 µL of ddH2O for dissolution and 10 µL of injection for processing by HPLC-Triple Quad 5500+ (AB SCIEX, USA). Chromatographic separation was achieved with a BioBasicTM AX (50 × 3 mm 5 µm, Thermofisher). The mobile phase was composed of solvent A (water: acetonitrile = 7:3, 10 mM ammonium acetate) and solvent B (water: acetonitrile = 7:3, 1 mM ammonium acetate; pH was adjusted to 10.0 with ammonia). Solvent gradient is as follows: 0–1.2 min, 95 % A; 1.2–2.3 min, 95 %-50 % A; 2.3–5.0 min, 50–0 % A; 5.0–6.0 min, 0–95 % A; 6.0–8.0, 95 % A.

### Statistical analyses

All statistical analyses were conducted utilizing GraphPad Prism 10.0 software. Comparisons between two groups were assessed by unpaired 2-tailed Student’s t-tests or Mann-Whitney test. Comparisons between multiple groups were performed using one-way (followed by Dunnett’s post hoc test), Kruskal-Wallis test (followed by Dunn’s post hoc test), or two-way ANOVA (followed by Bonferroni’s post hoc test). All *p* values are indicated in the figures, with specific statistical methods detailed in the corresponding figure legends.

## Results

### Intestinal PANK3 is downregulated in the colonic mucosa of IBD patients and murine colitis

**Fig. 1:**
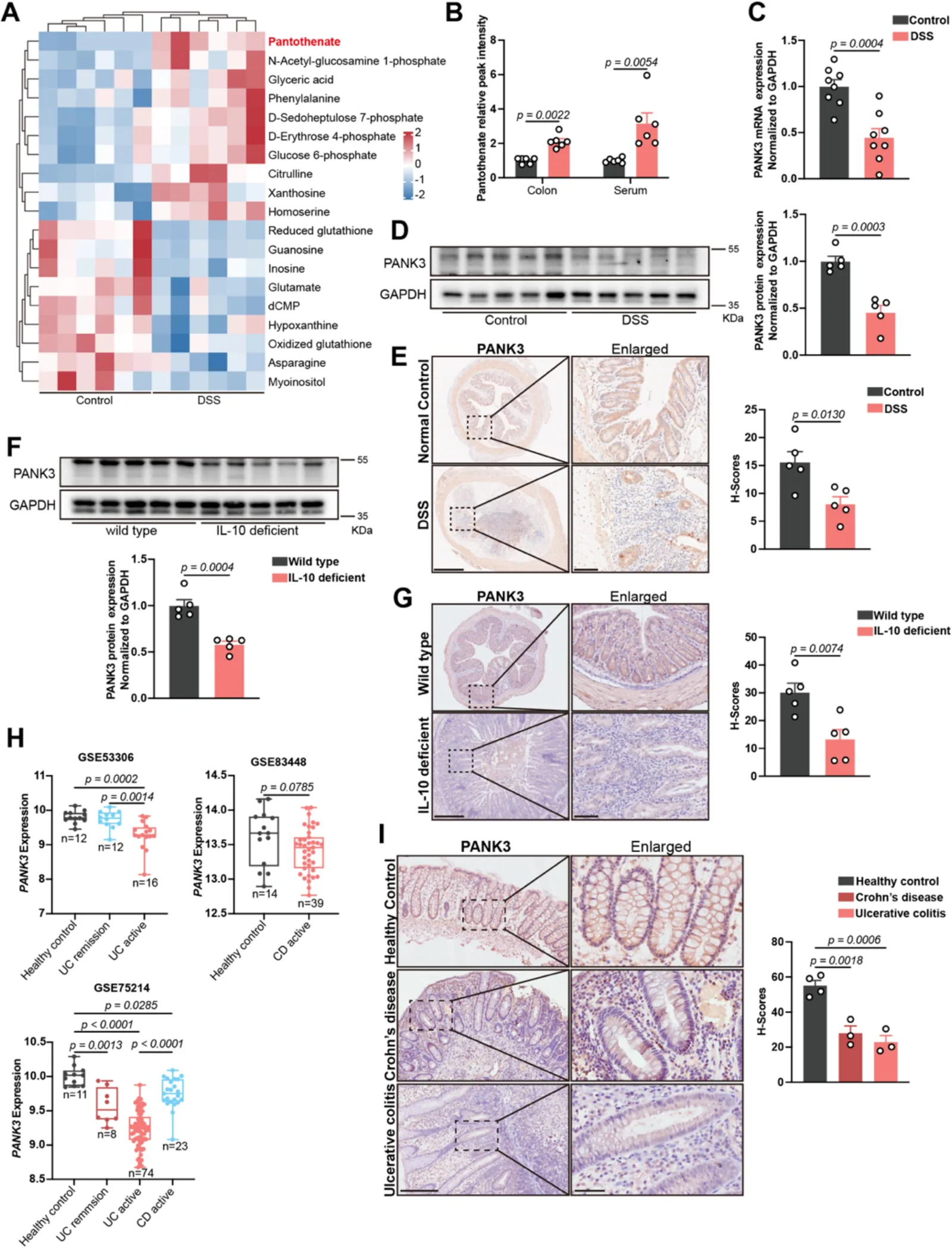
Colonic PANK3 is downregulated in IBD patients and murine colitis models. (A) Heatmap depicting colonic differential metabolites (p < 0.05) from control and DSS-induced colitis mice (n = 6). Data were Z-score normalized and subjected to hierarchical clustering. (B) Relative peak intensity of pantothenate in colon and serum (n = 6). (C–D) q-PCR (n = 8) (C) and Western blot (n = 5) (D) analysis of PANK3 in DSS-induced colitis. (E) Representative images and quantification of colonic PANK3 IHC staining (n = 5). Scale bars, 500 µm (left) and 100 µm (right). (F) Western blot analysis of PANK3 expression in IL-10^-/-^ mice (n = 5). (G) Representative images and quantification of IHC analysis of colonic PANK3 in IL-10^-/-^mice (n = 5). Scale bars, 500 µm (left) and 100 µm (right). (H) *PANK3* expression in the intestinal mucosal biopsies of healthy individuals and IBD patients from GEO datasets (GSE53306, GSE75214, and GSE83448). (I) Representative images and quantitative analysis of IHC for PANK3 on intestinal biopsies from healthy controls (n = 4), Crohn’s disease patients (n = 3), and ulcerative colitis patients (n = 3). Scale bars, 250 µm (left) and 50 µm (right). Data were expressed as mean ± SEM. *p* values were provided in figures and determined by unpaired Student’s *t*-test for B, C, D, E, F, G, and H (GSE83448), one-way ANOVA with Dunnett’s multiple comparisons for H (GSE53306 and GSE75214) and I.

To elucidate the potential relationship between endogenous metabolism and the progression of IBD, a systematic metabolomic analysis of DSS-induced experimental colitis model was initially conducted. A significant elevation in the pantothenate level was observed in both the colon and serum of the colitis mice compared with those of the normal controls (Fig. 1A–B and Fig.S1A), suggesting dysfunction of pantothenate metabolism. Pantothenate is the key substance utilized for the de novo synthesis of coenzyme A, which can be produced either through vanin-catalyzed hydrolysis or phosphorylated by pantothenate kinase (PANK) [25,26] (Fig.S1B). Although both upstream vanin and downstream PANK play key roles in the production of coenzyme A [27], the role of vanin in IBD pathogenesis is controversial. Therefore, this study focused on the phenotype and function of PANK. The data revealed that PANK3 expression exhibited significantly greater reduction than the other two isoforms of colonic PANK (PANK1 and PANK2) in DSS-induced colitis mice versus normal controls (Fig. 1C–D and Fig.S1C–F). Immunohistochemical staining confirmed this observation, directly showing decreased PANK3 levels within the colonic epithelium (Fig. 1E). Considering that a well-characterized mouse model of chronic colitis reproduces the clinical features of UC patients, an IL-10 knockout model of spontaneous colitis was established, in which the downregulation of PANK3 was further confirmed (Fig. 1F–G). Furthermore, analysis of intestinal biopsies from IBD patients in GEO datasets (GSE53306, GSE83448, GSE75214) revealed markedly decreased PANK3 expression in those with active CD or UC compared to inactive patients and healthy controls (Fig. 1H). This finding was corroborated at the tissue level, where a marked decrease in PANK3 was also observed in the intestinal epithelium of clinical CD and UC patients (Fig. 1I).

Consistently, qPCR and immunoblotting analysis revealed reduced expression of PANK3 in the human colon adenocarcinoma cell line HT29 and the colon epithelial cell line NCM460 after treatment with TNF-α and IFN-γ (Fig.S1G–I). Collectively, these findings suggest that downregulated intestinal epithelial PANK3 is a significant feature in the pathogenesis of UC.

### PANK3 regulates the integrity and function of the intestinal barrier *in vitro*

**Fig. 2:**
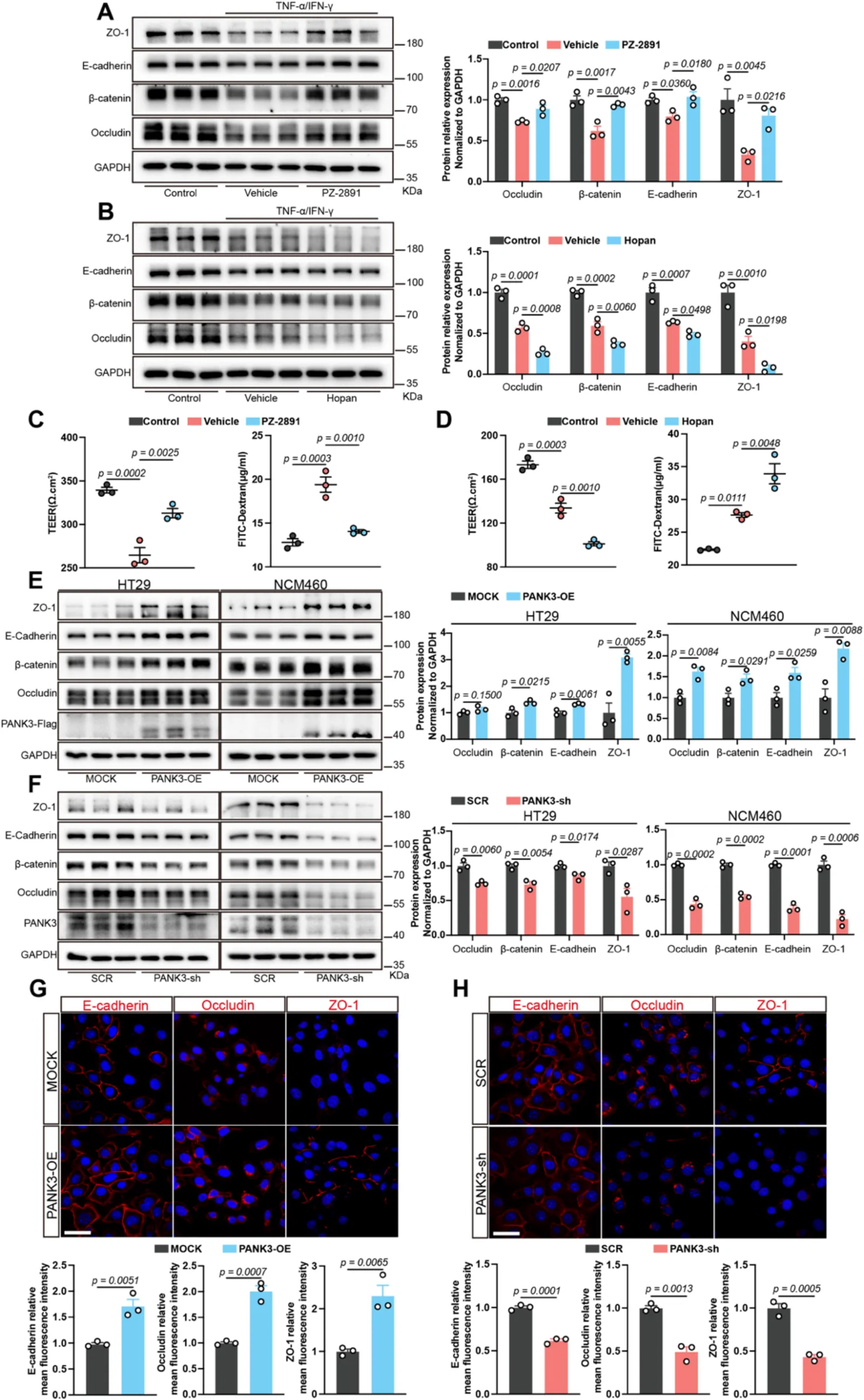
PANK3 improves epithelial barrier integrity in intestinal epithelial cell lines. (A–B) The expression of intercellular junctional proteins in HT29 cells treated with 30 ng/mL TNF-α/IFN-γ combined with 2 µM PZ-2891 (n = 3) (A) or 250 µM Hopan (n = 3) (B). (C–D) TEER and paracellular permeability of Caco-2 monolayer cells treated with 30 ng/mL TNF-α/IFN-γ combined with 2 µM PZ-2891 (n = 3) (C) or 250 µM (n = 3) Hopan (D). (E) The expression of intercellular junctional proteins in PANK3-overexpressing HT29 cells (left) or NCM460 cells (right) treated with 30 ng/mL TNF-α/IFN-γ (n = 3). (F) The expression of intercellular junctional proteins in PANK3-knocking down HT29 cells (left) or NCM460 cells (right) treated with 30 ng/mL TNF-α/IFN-γ (n = 3). (G–H) Immunofluorescence analysis of junction proteins with PANK3 modulation in TNF-α/IFN-γ treated PANK3-overexpressing (G) or PANK3-knocking down (H) NCM460 cells (n = 3). Scale bars, 50 µm. Data were expressed as mean ± SEM. *p* values were provided in figures and determined by one-way ANOVA with Dunnett’s multiple comparisons for A, B, C, and D, unpaired Student’s *t*-test for E, F, G, and H.

To identify the function of PANK3 in intestinal epithelial damage, a model induced by TNF-α and IFN-γ was established in HT29 cells. Administration of the PANK3 agonist PZ-2891 had a protective effect on barrier integrity, as evidenced by the marked upregulation of multiple intercellular junctional proteins (Fig. 2A). In contrast, the administration of Hopan, a competitive inhibitor of PANK, exacerbated the reduction in intercellular junctional proteins (Fig. 2B)

In addition, a Caco-2 monolayer cell model was employed and treated with PZ-2891 or Hopan. Transepithelial electrical resistance (TEER) and paracellular FD4 permeability determination revealed that PZ-2891 rescued the integrity of the barrier, whereas Hopan exacerbated barrier damage (Fig. 2C–D).

In addition to the agonist PZ-2891 and the inhibitor Hopan, direct genetic regulation of PANK3 was performed in HT29 and NCM460 cells. Overexpression of PANK3 rescued inflammation-induced barrier damage, whereas PANK3 knockdown had the opposite effect (Fig. 2E–F). Further validation of the safeguarding effects of PANK3 on barrier integrity was accomplished by using immunofluorescence staining (Fig. 2G–H). These results underscore the critical role of PANK3 in maintaining epithelial barrier function.

### PANK3 regulates the integrity and function of the intestinal barrier *in vivo*

To further determine the role of PANK3 in UC pathogenesis, DSS-treated mice were orally administered PZ-2891 (Fig.S2A). PZ-2891 treatment significantly reversed weight loss and colon shortening (Fig.S2B–C), followed by improvements in histological features, including preserved crypt architecture, maintenance of goblet cells, and diminished inflammatory cell infiltration (Fig.S2D). Consistently, the reduction in FD-4 leakage suggested enhanced intestinal barrier integrity (Fig.S2E). Additionally, the expression and distribution of key epithelial junctional proteins were markedly restored after PZ-2891 administration (Fig.S2F–G). Conversely, Hopan treatment exacerbated DSS-induced colon damage, as shown by extensive crypt loss, submucosal edema, and elevated barrier permeability, compared with vehicle controls (Fig.S2H–L). This pathological progression was correlated with decreased epithelial junctional protein expression (Fig.S2M–N).

**Fig. 3:**
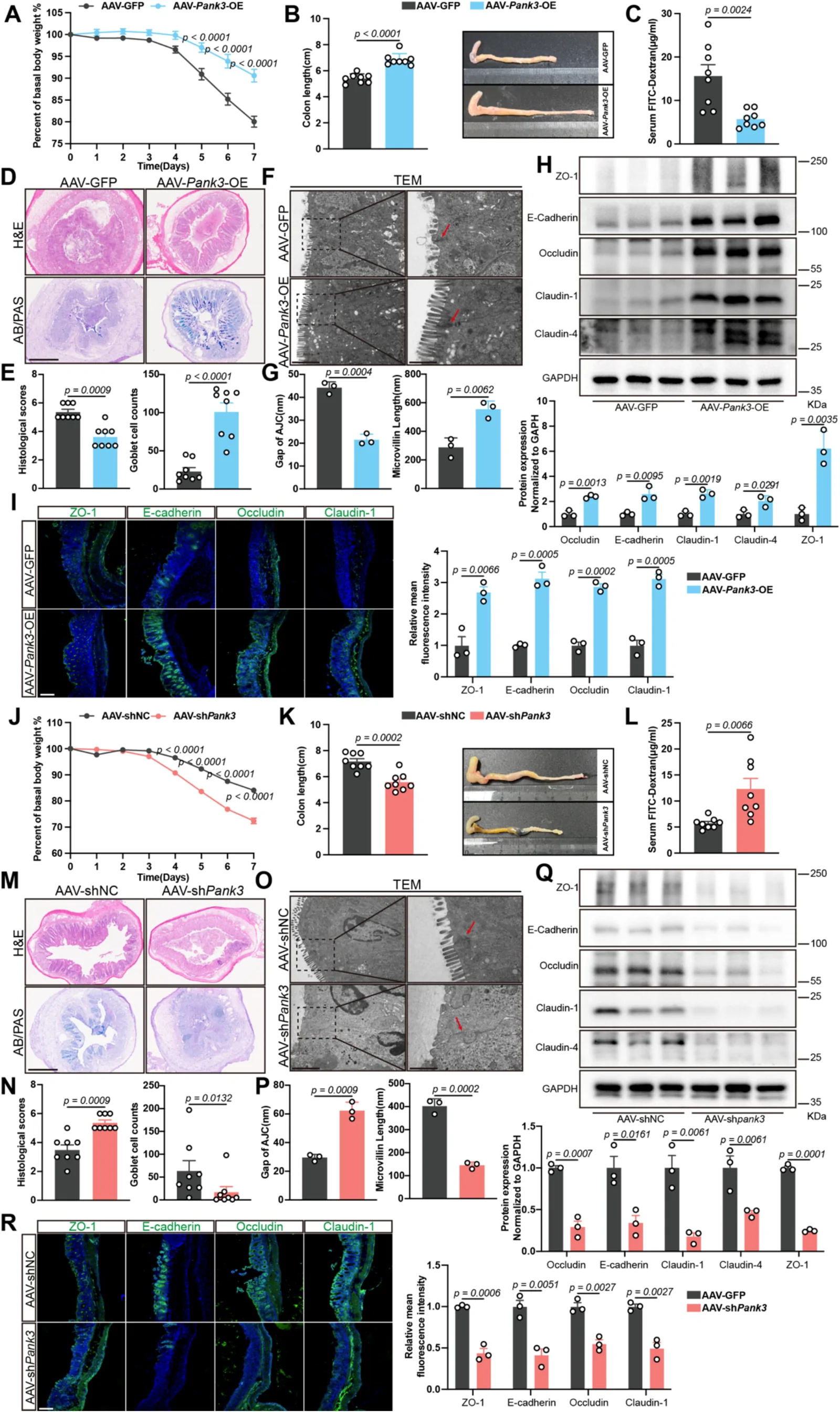
PANK3 confers protection against DSS-induced colitis and improves the intestinal barrier. (A–I) AAV-*Pank3*-OE and AAV-GFP mice were treated with DSS for 7 days. Monitoring of daily weight changes (n = 8) (A), colon length and representative images of mouse colon (n = 8) (B), serum FITC-dextran assays (n = 8) (C), representative histological H&E and AB-PAS staining images, scale bars, 500 µm (D), histological scores and goblet cells counts (n = 8) (E),representative TEM images of colonic mucosa, scale bars, 2 µm (left) and 1 µm (right) (F), measurement of AJC gaps and microvilli length (n = 3) (G), the expression of colonic intercellular junctional proteins analyzed by western blot (n = 3) (H), immunofluorescence analysis of colonic ZO-1, E-cadherin, Occludin, and Claudin-1 (n = 3), scale bars, 200 µm (I). (J–R) AAV-sh*Pank3* and AAV-shNC mice were treated with DSS for 7 days. Daily weight changes recording (n = 8) (J), colon length and colon images (n = 8) (K), serum FITC-dextran assays (n = 8) (L), histological H&E and AB-PAS staining, scale bars, 500 µm (M), histological scores and goblet cells counts (n = 8) (N), colonic mucosa TEM images, scale bars, 2 µm (left) and 1 µm (right) (O), measurement AJC gaps and microvilli length (n = 3) (P), the expression of intercellular junctional proteins analyzed by western blot (n = 3) (Q), colonic ZO-1, E-cadherin, Occludin, and Claudin-1 immunofluorescence analysis (n = 3), scale bars, 200 µm (R). Data were expressed as mean ± SEM. *p* values were provided in figures and determined by two-way ANOVA with Bonferroni’s multiple comparisons test for A and J, unpaired Student’s *t*-test for B, C, G, H, I, K, L, P, Q, and R, and Mann-Whitney test for E and N.

Given the potential for off-target effects with pharmacological modulation, intestinal-specific PANK3 overexpression via adeno-associated virus (AAV-*Pank3*-OE) was established, as confirmed by immunoblotting and immunofluorescence (Fig.S3A–C). When exposed to DSS stress, the weight loss, colon shortening, barrier integrity, and recovery of the crypt architecture, inflammatory infiltration as well as goblet cell population improved in AAV-*Pank3*-OE mice (Fig. 3A–E). Additionally, the shortened microvilli and disrupted apical junction complex (AJC) were rescued by PANK3 overexpression according to TEM analysis (Fig. 3F–G), as were the expression and localization of key junctional proteins (Fig. 3H–I). In contrast, PANK3 knockdown (AAV-sh*Pank3*, validated in Fig.S3D–F) exacerbated colitis-associated phenotypes and barrier damage (Fig. 3J–N). Additionally, the architectures of AJC and microvilli were extensively disrupted in AAV-sh*Pank3* mice (Fig. 3O–P), accompanied by a significant decrease in barrier-related proteins (Fig. 3Q–R). These results indicate that PANK3 plays a crucial role in ameliorating intestinal barrier injury caused by UC.

### PANK3 suppresses inflammation-induced epithelial‒mesenchymal transition

**Fig. 4:**
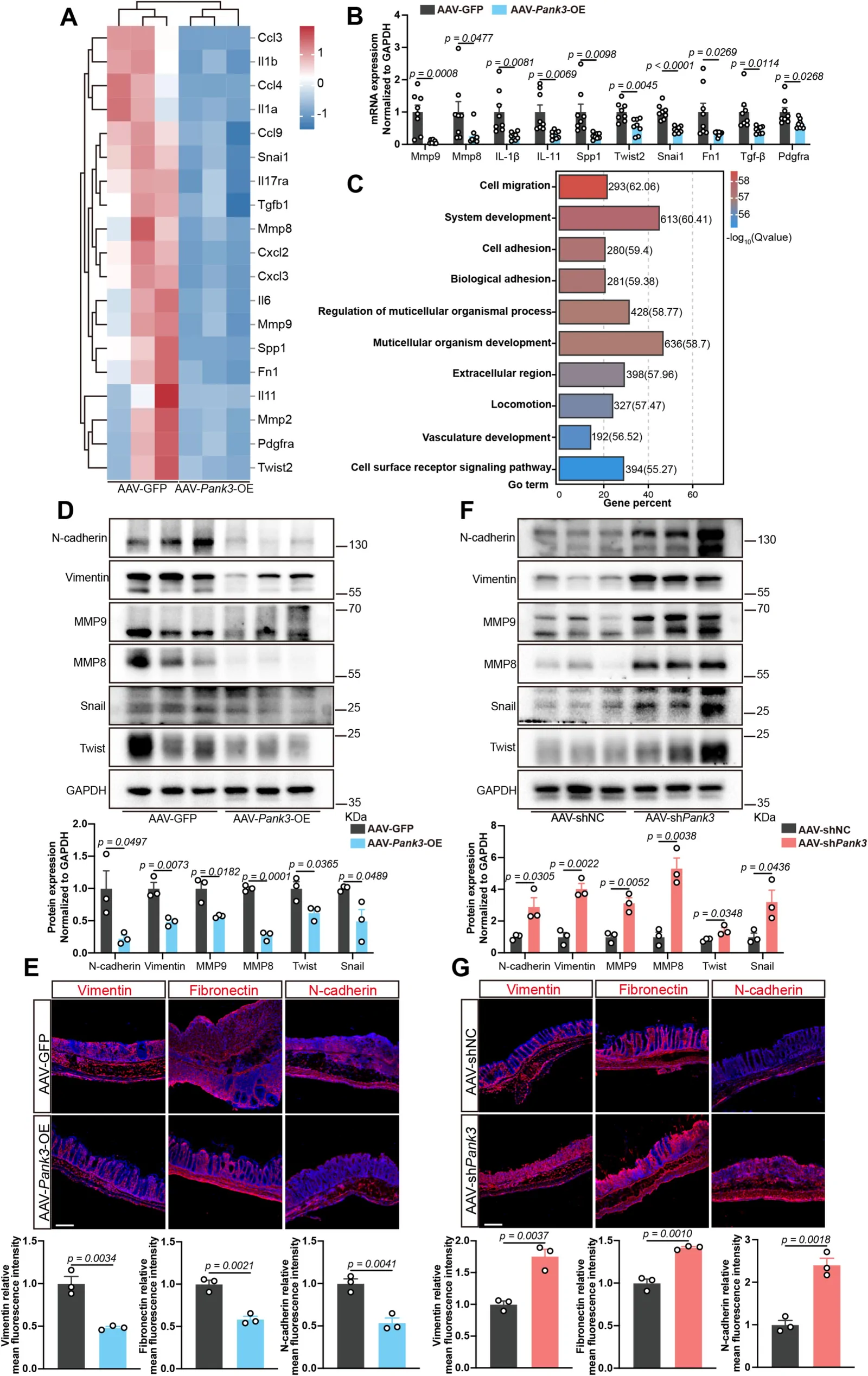
PANK3 represses EMT progression in DSS-induced colitis. (A) Heatmap of representative colonic DEGs (FDR < 0.05, log2|FC|>1) related with inflammation, ECM and EMT. Data were Z-score normalized and subjected to hierarchical clustering. The color scale represents expression levels. (B) Verification of representative DEGs by qRT-PCR (n = 8). (C) Gene ontology (GO) analysis of DEGs. (D) Expression of colonic EMT-related proteins in AAV-*Pank3*-OE mice analyzed by western blot (n = 3). (E) Immunofluorescence analysis of colonic vimentin, fibronectin, and N-cadherin from AAV-*Pank3*-OE mice (n = 3), scale bars, 200 µm. (F) Expression of colonic EMT-related proteins in AAV-sh*Pank3* mice analyzed by western blot (n = 3). (G) Immunofluorescence analysis of colonic vimentin, fibronectin, and N-cadherin from AAV-sh*Pank3* mice (n = 3), scale bars, 200 µm. Data were expressed as mean ± SEM. *p* values were provided in figures and determined by unpaired Student’s *t*-test.

Based on the findings that PANK3 regulates epithelial barrier function both *in vitro* and *in vivo*, the downstream mechanisms were investigated through transcriptomic analysis of colonic tissues from DSS-treated AAV-GFP and AAV-*Pank3*-OE mice. Significant downregulation of multiple gene clusters, including proinflammatory cytokines (e.g., Ils, Ccls and Cxcls), extracellular matrix (ECM) components (e.g., MMPs, Fn1 and Spp1), and epithelial‒mesenchymal transition (EMT)-related genes (e.g., Snai1 and Twist), was identified via comparative analysis (Fig. 4A), with some of the representative differential genes validated via qPCR (Fig. 4B). Gene Ontology (GO) analysis characterized the alterations in cell migration, adhesion, locomotion, and ECM remodeling (Fig. 4C). All the above hallmark features are deeply involved in EMT, a pervasive biological process during which epithelial cells suffer from reduced intercellular junctions and increased cell motility [28]. Hence, PANK3 was hypothesized to restore epithelial integrity by repressing EMT. Notably, PANK3 overexpression inhibited EMT through the downregulation of mesenchymal markers, MMPs, and EMT-driving transcription factors, synergistically restoring aberrant extensive distribution of mesenchymal markers (Fig. 4D–E). In contrast, PANK3 depletion triggered the coordinated upregulation of EMT hallmarks (Fig. 4F–G), indicating that reduced PANK3 contributes to intestinal barrier impairment through the progression of EMT in UC.

### PANK3 inhibits the MAPK and PI3K/AKT pathways and suppresses EMT by enhancing the synthesis of free CoA

**Fig. 5:**
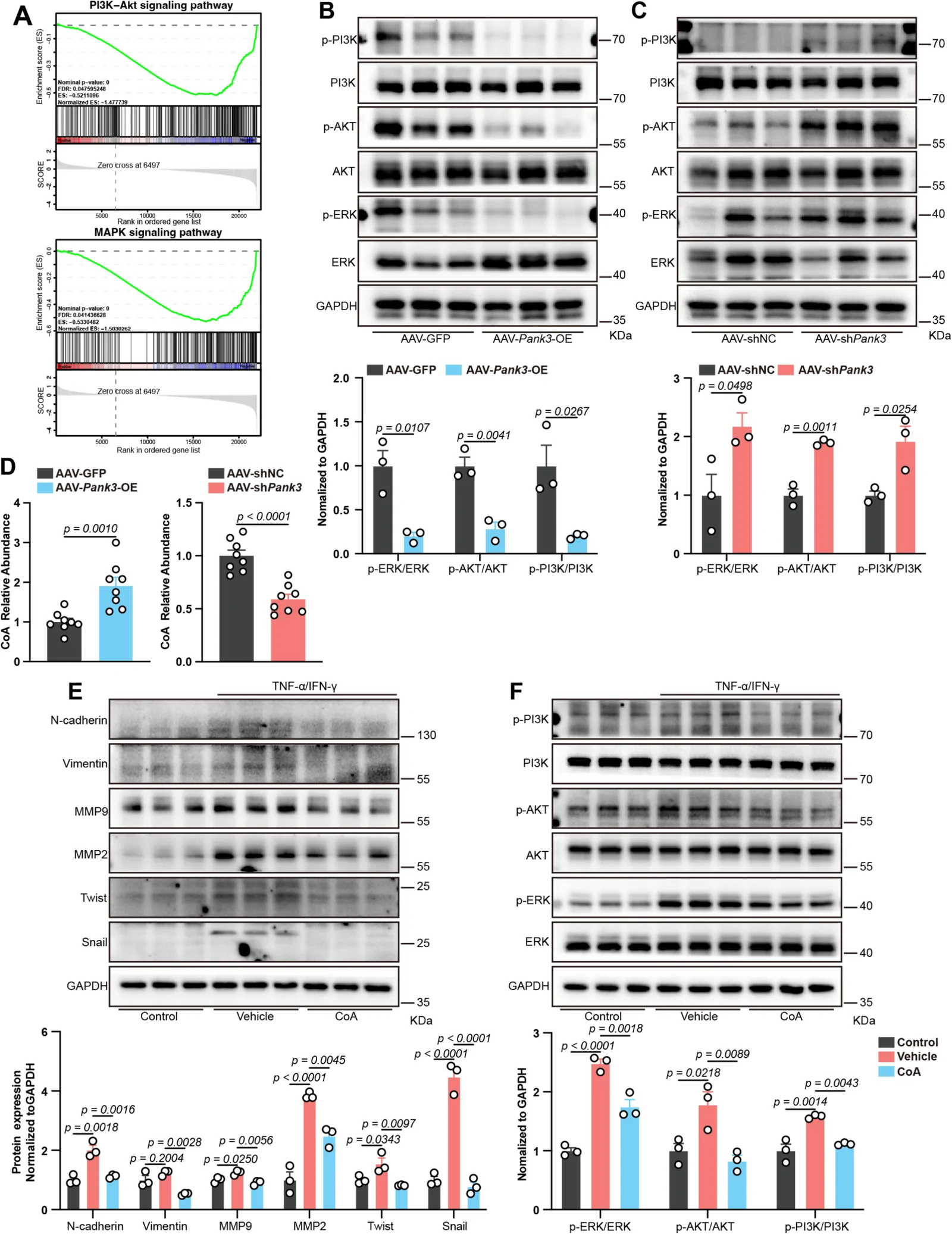
PANK3 promotes the production of free coenzyme A, thereby inhibiting the PI3K/AKT and MAPK pathways and repressing EMT. (A) GSEA analysis revealed enrichment of PI3K/AKT and MAPK pathways. (B–C) Phosphorylation level of PI3K, AKT and ERK in AAV-*Pank3*-OE (n = 3) (B) and AAV-sh*Pank3* mice (n = 3) (C). (D) Colonic free CoA relative abundance fold changes after PANK3 overexpressing (n = 8) (left) and knocking down (n = 8) (right). (E–F) The expression of EMT-related proteins (E) and phosphorylation level of PI3K, AKT and ERK (F) (n = 3) in HT29 cells treated with 30 ng/mL TNF-α/IFN-γ combined with 250 µM CoA. Data were expressed as mean ± SEM. *p* values were provided in figures and determined by unpaired Student’s *t*-test for B, C, and D, one-way ANOVA with Dunnett’s multiple comparisons for E and F.

Following the discovery that PANK3 inhibits EMT, the underlying molecular mechanisms were further investigated using gene set enrichment analysis (GSEA). In AAV-*Pank3*-OE mice, the typical EMT-associated pathways of the PI3K/AKT and MAPK signaling cascades were identified as the pathways associated with the most significantly suppressed genes (Fig. 5A). Consistently, the phosphorylation of PI3K, AKT and ERK was markedly decreased in AAV-*Pank3*-OE mice (Fig. 5B), whereas AAV-sh*Pank3* mice exhibited concomitant increases in these activation markers (Fig. 5C). Based on the consistent effects observed from both pharmacological and genetic regulation of PANK3, its functional role was inferred to depend on its metabolic enzyme activity. This hypothesis was subsequently tested through complete pantothenate deprivation *in vitro*, which largely abolished the barrier protection induced by either PANK3 agonism or overexpression (Fig.S4A–B), thereby providing initial experimental validation. Furthermore, given the central role of PANK in the CoA biosynthetic pathway and, particularly, emerging evidence supporting the potential role of CoA in IBD [29,30], a quantitative LC-MS/MS assay of intestinal CoA was conducted following PANK3 genetic modulation. The results demonstrated that PANK3 overexpression significantly increased CoA abundance, whereas its knockdown conversely led to a reduction (Fig. 5D). To further validate that CoA mediates the downstream effects of PANK3, exogenous CoA was supplemented *in vitro*, with results showing that CoA significantly suppressed the TNF-α/IFN-γ-induced EMT progression and activation of the PI3K/AKT and MAPK pathways (Fig. 5E–F). Moreover, CoA supplementation effectively reversed the barrier impairment and EMT activation phenotypes induced by PANK3 knockdown (Fig.S4C–D). Overall, these data suggest that upregulation of PANK3 inhibits the MAPK and PI3K/AKT signaling cascades and suppresses activated EMT by enhancing CoA synthesis.

### c-Myc is the key transcription factor regulating PANK3

**Fig. 6:**
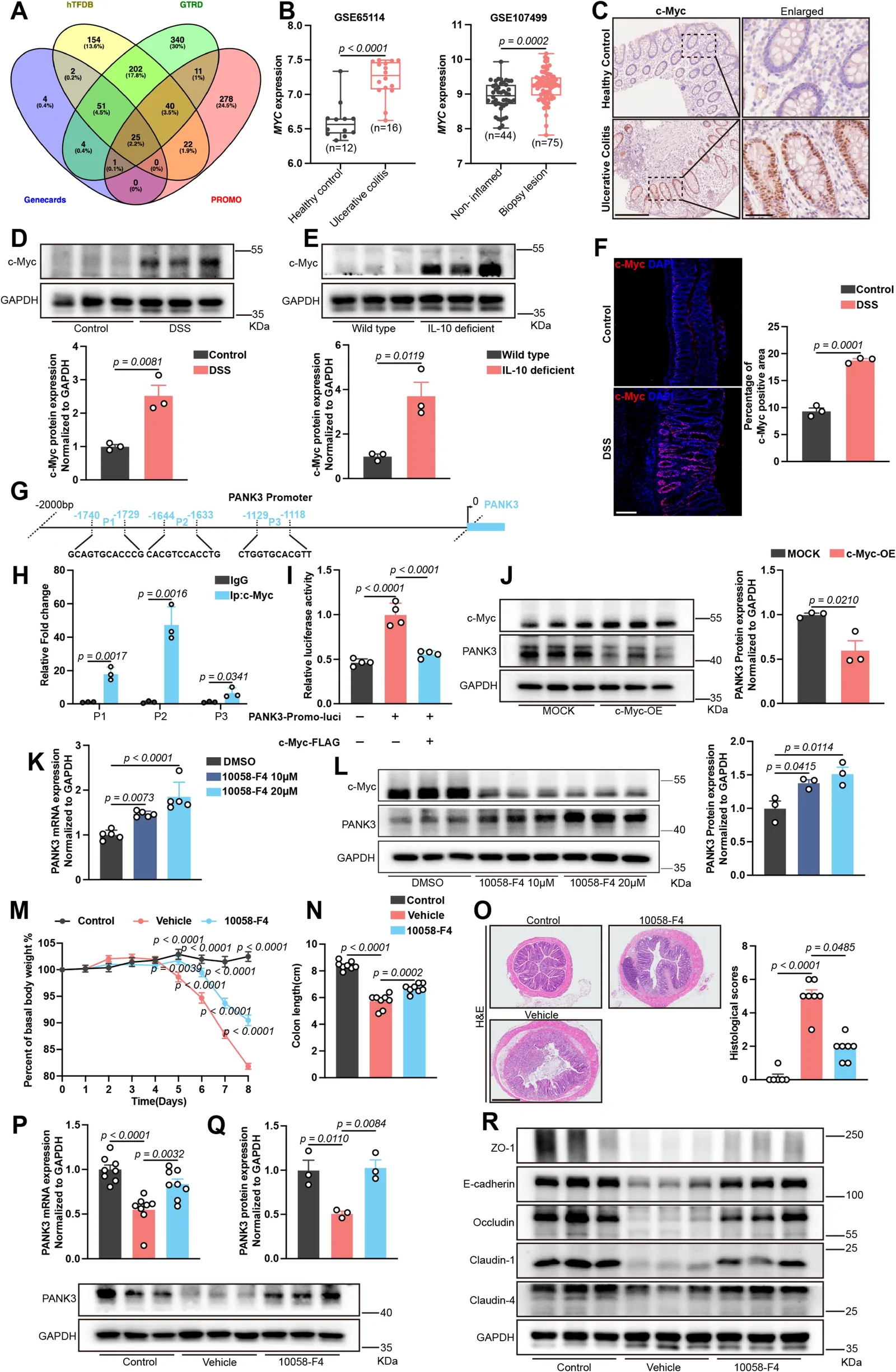
Inflammation-driven c-Myc transcriptionally suppresses *Pank3*. (A) Venn plot of potential TFs of *Pank3* according to four TFs prediction databases. (B) c-Myc expression in the mucosal biopsies of UC patients from GEO datasets (GSE65114, GSE107499). (C) Representative images of c-Myc IHC staining from UC patients. Scale bars, 250 µm (left) and 50 µm (right). (D–E) The expression of colonic c-Myc in DSS-induced colitis (n = 3) (D) and IL-10 deficient mice (n = 3) (E). (F) Immunofluorescence analysis of c-Myc in colon sections from DSS-induced colitis (n = 3). Scale bars, 200 µm. (G) Potential binding motifs of c-Myc in the promoter region of *Pank3* gene according to JASPAR database. (H) Verification of binding between c-Myc and *Pank3* promoter by ChIP-qPCR assay (n = 3). (I) Dual-luciferase reporter gene assay (n = 4). (J) Western blot analysis of PANK3 expression in c-Myc overexpressing HT29 cells (n = 3). (K–L) qRT-PCR (n = 5) (K) and western blot analysis (n = 3) (L) of PANK3 expression in HT29 cells treated with 10 µM and 20 µM of 10058-F4. (M−R) DSS-induced colitis mice were administrated with 10058-F4. Monitoring of weight loss (n = 8) (M), colon length (n = 8) (N), histological H&E staining and histological scores (n = 6–7), scale bars, 500 µm (O), qRT-PCR analysis of colonic PANK3 (n = 8) (P), western blot analysis of PANK3 (n = 3) (Q), western blot analysis of intercellular junctional proteins (n = 3) (R). Data were expressed as mean ± SEM. *p* values were provided in figures and determined by unpaired Student’s *t*-test for B, D, E, F, H, and J, one-way ANOVA for with Dunnett’s multiple comparisons for I, K, L, P, and Q, two-way ANOVA with Bonferroni’s multiple comparisons for M, and Kruskal-Wallis test with Dunn’s multiple comparisons for O.

The upstream regulator of PANK3 was investigated to explore the underlying mechanisms of PANK3 downregulation. A reduction in PANK3 mRNA levels was detected in both UC patients and mice with colitis, indicating the suppression of PANK3 transcription. Thus, public prediction databases for potential transcription factor (TF) were interrogated, and c-Myc emerged as a prime candidate showing significant upregulation in the colonic lesions of UC patients, which was further confirmed by IHC staining (Fig. 6A–C). Consistently, increases in c-Myc were observed both *in vivo* and *in vitro*, i.e., in DSS-induced and IL-10-knockout mice (Fig. 6D–F) and in TNF-α/IFN-γ-treated HT29 and NCM460 cell lines (Fig.S5A–D).

The 2000 bases before the *Pank3* gene coding sequences were subsequently screened on the JASPAR website to examine possible binding sites of c-Myc. A total of three putative binding sequences were identified (Fig. 6G), and their direct binding effect was validated through chromatin immunoprecipitation and qPCR (ChIP‒qPCR) (Fig. 6H). The transcriptional effects of c-Myc on PANK3 were evaluated via a dual-luciferase reporter assay in HEK-293T cells transfected with the full-length *Pank3* promoter. Overexpression of c-Myc led to a marked decreased in the activity of the *Pank3* promoter (Fig. 6I) and resulted in the downregulation of PANK3 (Fig. 6J). In contrast, the administration of 10058-F4, a specific c-Myc inhibitor, increased both the mRNA and protein expression levels of PANK3 in a dose-dependent manner *in* vitro (Fig. 6K–L and Fig.S5E). Furthermore, pharmacological inhibition of c-Myc significantly ameliorated DSS-induced colitis and improved weight loss, colon shortening, and histological damage (Fig. 6M−O). This intervention restored PANK3 expression (Fig. 6P–Q) and improved intestinal barrier integrity through the upregulation of junctional proteins (Fig. 6R). In summary, c-Myc is the key transcription factor regulating the expression and function of PANK3, and the c-Myc-PANK3 axis plays a key role in the intestinal barrier disruption.

### Folic acid is a potential PANK3 agonist that promotes CoA synthesis and ameliorates colitis by improving intestinal barrier integrity

**Fig. 7:**
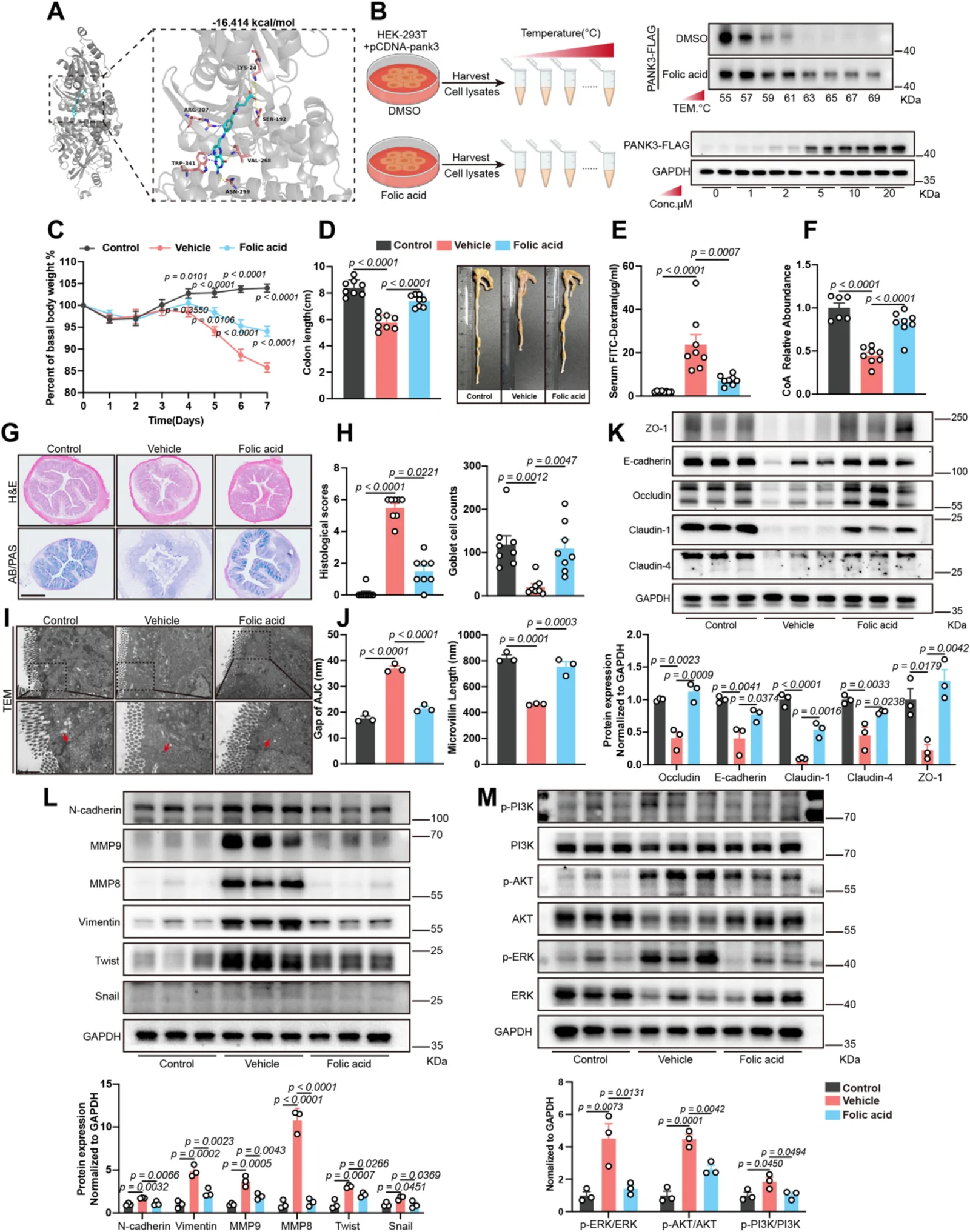
Folic acid functions as an agonist of PANK3 and ameliorates DSS-induced colitis. (A) Predicted binding pattern of folic acid to PANK3. H-bond (yellow) and π-π Stacking (blue) (B) Experiment diagram and immunoblots from CETSA showing stabilization of PANK3 by folic acid under different temperatures (upper) and concentrations (down). (C–M) DSS-induced colitis mice were treated with folic acid. Recording of daily weight (n = 8) (C), colon length and representative colon image (n = 8) (D), serum FITC-dextran assay (n = 8) (E), determination of relative abundance of colonic CoA (n = 6–8) (F), histological H&E and AB-PAS staining, scale bars, 500 µm (G), histological scores and goblet cell counts (n = 8) (H), representative TEM images of colonic mucosa, scale bars, 2 µm (upper) and 1 µm (down) (I), measurement of AJC gaps and microvilli length (right) (n = 3) (J), expression of intercellular junctional proteins analyzed by western blot (n = 3) (K), expression of EMT-related proteins (n = 3) (L), phosphorylation of PI3K, AKT and ERK (n = 3) (M). Data were expressed as mean ± SEM. *p* values were provided in figures and determined by two-way ANOVA for with Bonferroni’s multiple comparisons for C, one-way ANOVA with Dunnett’s multiple comparisons for D, E, F, J, K, L, and M, and Kruskal-Wallis test with Dunn’s multiple comparisons for H.

High-throughput virtual screening of small molecules identified folic acid, a naturally derived vitamin, as the top agonist of PANK3. Molecular docking revealed the key binding residues Lys24, Ser192, Arg207, Val268, Asn299, and Trp341 (Fig. 7A). A cellular thermal shift assay (CETSA) confirmed the binding of folic acid to PANK3, demonstrating enhanced thermal stability and a dose-dependent stabilization (Fig. 7B).

The therapeutic potential of folic acid was assessed in a DSS-induced UC model. After oral administration of folic acid at 30 mg/kg, there were distinct improvements in the pathological features of UC, including progressive weight loss, colon length reduction, and intestinal barrier disruption (Fig. 7C-E). Importantly, folic acid significantly stimulated the function of PANK3, elevated colonic CoA levels (Fig. 7F) and restored epithelial architecture, as evidenced by the increase in crypt integrity, maintenance of goblet cells, and reduction in inflammatory infiltration (Fig. 7G-H). Ultrastructural analysis further demonstrated improved microvillus organization and AJC restoration (Fig. 7I-J).

In addition, as indicators of intestinal integrity, epithelial junctional proteins were upregulated (Fig. 7K), along with decreased expression of mesenchymal markers, MMPs, and EMT regulators (Fig. 7L), after folic acid administration. Furthermore, activation of PI3K/AKT and MAPK pathways was suppressed (Fig. 7M). Consistently, *in vitro* experiments confirmed the barrier-protective effects of folic acid; however, this protection was abolished upon PANK3 knockdown, demonstrating its PANK3 dependence (Fig.S6A). Collectively, these findings demonstrate that folic acid functions as a potential agonist of PANK3 that can improve intestinal barrier integrity and ameliorate DSS-induced colitis.

## Discussion

By analyzing metabolic patterns in DSS-induced colitis mice via nontargeted metabolomics, a significant increase in the level of colonic pantothenate was initially observed, indicating that the pantothenate metabolism pathway was dysregulated. This finding directed our investigation toward PANKs, the downstream enzymes responsible for pantothenate phosphorylation. Through the integration of public transcriptomic datasets with experimental validation across distinct cellular and murine colitis models and clinical patients, a specific downregulation of PANK3, the predominant intestinal isoform [31], was identified at both the mRNA and protein levels. Moreover, although previous studies have established the pathological overexpression of vanin, an upstream enzyme responsible for pantothenate liberation, in IBD-inflamed mucosa and its therapeutic implications [32,33], emerging evidence has paradoxically suggested protective effects of IEC-specific vanin overexpression in DSS-induced colitis [30]. Independent of the unsolved controversial role of vanin, our study identified epithelial-specific PANK3 deficiency as a distinct pathological feature of UC. This epithelium-restricted PANK3 downregulation is associated with epithelial damage, suggesting its potential involvement in maintaining intestinal epithelial barrier integrity.

Comprehensive functional validation employing pharmacological modulators and genetic interventions in cellular and murine models demonstrated the protective role of PANK3 in UC, as the upregulation of PANK3 increased intestinal barrier integrity and ameliorated disease progression, whereas its downregulation exacerbated epithelial damage and increased the severity of colitis. Mechanistically, PANK3 maintains intestinal epithelial homeostasis through the regulation of CoA biosynthesis, a prerequisite for normal epithelial cell function. Importantly, pantothenate supplementation alleviated colitis via CoA-dependent metabolic reprogramming of TH17 cells, confirming the crucial role of the CoA synthesis pathway in the intestinal inflammatory microenvironment [29]. Collectively, these findings suggest that PANK3 is a druggable target for restoring barrier integrity in UC pathogenesis.

Epithelial cells form polarized architectures through apical junction complexes, including tight junctions and adherens junctions, coupled with basement membrane anchoring via the extracellular matrix [34]. These integrated structural systems are critical for maintaining apical–basal polarity and epithelial barrier integrity [35]. During pathological EMT, coordinated cytoskeletal reorganization triggers dissolution of junctional complexes, characterized by marked downregulation of junction proteins (e.g., ZO-1, E-cadherin, and claudins) [36], accompanied by basement membrane degradation, directly driving barrier dysfunction [37,38]. While EMT facilitates epithelial restitution following acute injury, its pathological persistence under chronic inflammation paradoxically drives progressive epithelial damage, culminating in tissue degeneration and fibrotic remodeling [39]. Correspondingly, clinical IBD mucosal samples exhibit this overactive EMT signature, which is correlated with disease severity and therapeutic resistance, underscoring its detrimental role in disease progression [40,41].

Despite the complex network of upstream regulatory mechanisms involved in EMT, our study identified PANK3 as a potent metabolic brake of this pathogenic cascade. In a DSS-induced colitis model, overexpression of PANK3 significantly inhibited the upstream EMT-inducing MAPK and PI3K/AKT signaling pathways, resulting in decreased expression of the EMT-related transcription factors Snail and Twist. This coordinated inhibition blocked MMP-mediated extracellular matrix remodeling and reversed the expression of mesenchymal transition markers (Vimentin and N-cadherin) while preserving junctional complex integrity via the upregulation of Occludin, ZO-1, Claudins, and E-cadherin. Crucially, the reconstituted junctional complex architecture directly demonstrated functional restoration of the epithelial barrier. These findings collectively validate the targeting of PANK3 as a novel strategy for counteracting EMT-driven barrier disruption in UC therapeutics.

While this study establishes the essential role of PANK3 in preserving intestinal barrier integrity and ameliorating UC, the upstream regulators governing its expression remain undefined. To this end, a systematic analysis of the transcription binding sites in the Pank3 promoter was performed, which identified c-Myc as a putative regulator. Although c-Myc is classically defined as a proto-oncogene[42], its involvement in inflammatory signaling cascades within non-cancerous diseases has been reported in emerging studies [[43], [44], [45]]. Consistently, c-Myc expression was elevated in colonic tissues from both UC patients and experimental colitis models, inversely correlating with PANK3 downregulation. This potential negative transcriptional regulation of PANK3 by c-Myc was further confirmed by ChIP and dual-luciferase reporter gene assays. *In vivo* functional validation revealed that pharmacological inhibition of c-Myc rescued PANK3 expression, concurrently suppressing EMT progression and enhancing intestinal barrier integrity in DSS-induced colitis model mice. Furthermore, the established oncogenic propensity of c-Myc, combined with its newly identified role in suppressing PANK3 during colitis, suggests a plausible mechanistic link between chronic inflammation and colorectal carcinogenesis. In support of these findings, decreasing PANK3 was identified as a diagnostic biomarker for early-stage colorectal cancer via bioinformatic analysis [46]. Taken together, the results of this study reveal that the c-Myc-PANK3 axis is a crucial regulator of intestinal barrier integrity in UC, where inflammation-driven c-Myc overexpression transcriptionally suppresses PANK3 to exacerbate barrier dysfunction. Targeting this axis restores barrier homeostasis, further highlighting the therapeutic potential of PANK3.

While our findings suggest a mechanistic connection between the PANK3/CoA axis and EMT, the scope of global transcriptomics and the potential non-specificity of AAV-mediated gene modulation across tissues and cell types limit the resolution of PANK3′s downstream effects, underscoring the need to further elucidate the precise biological effects of PANK3. Moreover, the mechanism by which PANK3-driven CoA synthesis suppresses the PI3K/AKT and MAPK pathways in inflammation warrants dedicated investigation, particularly since the PI3K/AKT pathway itself is known to promote *de novo* CoA synthesis under physiological conditions [26], suggesting a potential bidirectional relationship. Furthermore, beyond its central metabolic functions, CoA serves as an essential donor for key post-translational modifications such as 4′-phosphopantetheinylation and protein CoAlation [25], suggesting plausible non-metabolic routes through which it may exert regulatory influence. In parallel, the potential for PANK3 to possess non-catalytic functions, such as through protein–protein interactions, remains another direction for future investigation. Finally, building upon our evidence suggesting folic acid is a PANK3 agonist, future work should definitively establish its binding affinity through biophysical assays and demonstrate target-dependent efficacy in intestinal PANK3-KO mice.

Despite these limitations, our findings support the potential of PANK3 as a therapeutic target for UC and the future development of small-molecule agonists with enhanced potency, gut-restricted exposure, and an optimized safety profile. Alternatively, folic acid supplementation represents a safe and clinically accessible intervention. Notably, a *meta*-analysis of clinical studies reinforces this perspective, demonstrating that higher folate levels are a protective factor associated with a reduced risk of IBD [47], thereby providing clinical relevance to our mechanistic findings. While the therapeutic dose employed in our preclinical model exceeds typical clinical supplementation levels, this was necessary to overcome the pharmacokinetic limitations of conventional oral administration, particularly the low colonic distribution of systemically delivered folic acid [48]. Future clinical translation could further enhance efficacy and safety through optimized formulations such as enteric-coated tablets or colon-targeted delivery systems, which would increase local exposure while permitting lower systemic doses. Beyond its therapeutic implications, our data suggest circulating pantothenate/CoA levels and intestinal PANK3 expression may serve as clinically accessible biomarkers for aiding diagnosis and monitoring therapeutic efficacy. Nevertheless, translating this potential into clinical practice must await validation in larger, multi-center cohorts.

## Conclusion

In summary, this study establishes the c-Myc-PANK3 axis as central to intestinal epithelial barrier impairment in ulcerative colitis. Specifically, inflammation triggers c-Myc-mediated transcriptional repression of PANK3, leading to disrupted CoA synthesis and initiation of the EMT process via both PI3K/AKT and MAPK signaling pathways. Restoration of PANK3 expression and function effectively ameliorates intestinal barrier dysfunction and experimental colitis. These findings highlight PANK3 as a novel therapeutic target for UC, and support the clinical translation of its identified agonist, folic acid.

## Compliance with ethics requirements

All experiments involving animals were carried out in compliance with the ethical guidelines and protocols approved by the Ethical Committee for Experimental Animal Care of China Pharmaceutical University (Approval no: 2023–01-012).

## Declaration of competing interest

The authors declare that they have no known competing financial interests or personal relationships that could have appeared to influence the work reported in this paper.
