# Supplementary material for: c-Myc-PANK3-EMT axis regulates the structure and function of intestinal barrier in ulcerative colitis
Source: J Adv Res. 2025 Dec 8;87:931–46. doi: 10.1016/j.jare.2025.12.007 (PMC13539140; doi:10.1016/j.jare.2025.12.007)
Supplement: Supplementary file 1 [file mmc1.docx]

**Supplemental Figures**

**Fig.S1** Colonic PANK3, not PANK1 or PANK2, is downregulated in ulcerative colitis. (A) Heatmap of serum differential metabolites (FDR<0.05) analyzed by metabolomics (n=6). Data were Z-score normalized and subjected to hierarchical clustering. (B) The pantothenate metabolism and CoA biosynthesis pathway. (C-D) qRT-PCR analysis of PANK1 (n=8) (C) and PANK2 (n=8) (D) in DSS-induced colitis. (E-F) Western blot analysis of PANK1 (n=6) (E) and PANK2 (n=6) (F) in DSS-induced colitis. (G) qRT-PCR analysis of PANK3 in HT29 (n=6) (left) and NCM460 (n=6) (right) cells treated with 30 ng/mL TNF-α/IFN-γ. (H-I) Western blot analysis of PANK3 in HT29 (n=3) (H) and NCM460 (n=3) (I) cells treated with 30 ng/mL TNF-α/IFN-γ. Data were expressed as mean ± SEM. *p* values were provided in figures and determined by unpaired Student’s t-test.

**Fig.S2** Pharmacological agitation of PANK3 alleviates DSS-induced colitis, whereas its inhibition exacerbates colitis. (A-G) Mice were treated with vehicle or PZ-2891 during DSS induction. Experiment design diagram (A), daily weight loss record (n=8) (B), colon length and representative colon image (n=8) (C), representative images of colonic sections stained with H&E and AB-PAS, scale bars, 500 µm (D), serum FITC-dextran assay (n=8) (E), immunofluorescence images of ZO-1, E-cadherin, Occludin, Claudin-1, and Claudin-4, scale bars, 200 µm (F), western blot analysis of intercellular junctional proteins (n=3) (G). (H-N) Mice were treated with vehicle or Hopan during DSS induction. Experiment design diagram (H), daily weight loss record (n=8) (I), colon length and representative colon image (n=8) (J), representative images of colonic sections stained with H&E, scale bars, 500 µm (K), serum FITC-dextran assay (n=7) (L), immunofluorescence images of ZO-1, E-cadherin, Occludin, and Claudin-1, scale bars, 100 µm (M), western blot analysis of intercellular junctional proteins (n=3) (N). Data were expressed as mean ± SEM. *p* values were provided in figures and determined by two-way ANOVA with Bonferroni’s multiple comparisons for B and I, unpaired Student’s t-test for C, E, G, J, L, and N.

**Figure S3** Verification of *in vivo* colonic PANK3 overexpression and knockdown. (A) Experimental flow chart of colonic PANK3 overexpression. (B) Expression of colonic PANK3 analyzed by immunoblots (n=6). (C) Immunofluorescence analysis colonic PANK3 in AAV-GFP and AAV-*Pank3*-OE mice (n=3). Scale bars, 100 µm. (D) Experimental flow chart of colonic PANK3 knocking down. (E) Expression of colonic PANK3 analyzed by immunoblots (n=5). (F) Immunofluorescence analysis colonic PANK3 in AAV-shNC and AAV-sh*Pank3* mice. Scale bars, 100 µm. Data were expressed as mean ± SEM. *p* values were provided in figures and determined by unpaired Student’s t-test.

**Fig.S4** PANK3-mediated barrier protection requires its metabolic activity and CoA reverses the consequences of PANK3 deficiency. (A-B) Western blot analysis of barrier-associated proteins in pantothenate-deprived cells following PZ-2891 treatment (A) or PANK3 overexpression (B). (C-D) Exogenous CoA reverses the consequences of PANK3 deficiency, restoring barrier integrity protein levels (C) and inhibiting EMT marker expression (D) (n=3). Data were expressed as mean ± SEM. *p* values were provided in figures and determined by one-way ANOVA with Dunnett’s multiple comparisons for C and D.

**Fig.S5** c-Myc is upregulated in intestinal epithelial cells following TNF-α and IFN-γ treatment. (A-B) Western blot analysis of c-Myc expression in HT29 (A) and NCM460 cells (B) treated with 30 ng/mL TNF-α/IFN-γ (n=3). (C) Subcellular fractionation analysis of c-Myc in the cytoplasmic and nuclear fractions of HT29 cells under the same treatment. (D) Immunofluorescence analysis confirming the localization and expression of c-Myc in treated HT29 cells (n=3). Scale bars, 20 µm. (E) Western blot analysis of PANK3 in NCM460 cells following treatment with the c-Myc inhibitor 10058-F4 (n=3). Data were expressed as mean ± SEM. *p* values were provided in figures and determined by unpaired Student’s t-test for A, B, and D, one-way ANOVA with Dunnett’s multiple comparisons for E.

**Fig.S6** PANK3 knockdown reverses folic acid-induced barrier protection *in vitro*. (A) Effect of 1 µM folic acid on the expression of barrier-associated proteins in HT29 cells with scrambled control (SCR) or PANK3 knockdown, assessed by Western blot (n=6). Data were expressed as mean ± SEM. *p* values were provided in figures and determined by one-way ANOVA with Dunnett’s multiple comparisons.

**Supplemental Materials and Methods**

**LC-Q/TOF MS-based metabolomics assays**

50 µL serum sample was extracted by 200 µL 100% methanol (containing 10 µg/mL 4-Chloro-DL-phenylamine, IS); 20 mg colon sample was extracted by 800 µL 80% methanol (containing 10 µg/mL 4-Chloro-DL-phenylamine, IS). After homogenization and centrifugation, 200 µL of supernatant was dried by vacuum centrifugation, followed by redissolving with 100 µL ddH2O and 10 µL sample was subjected to metabolomics analysis by HPLC Triple TOF/MS 5600 system (AB SCIEX, USA). An equal volume of each prepared sample was pooled to create a quality control (QC) sample that was analyzed throughout the sequence to monitor instrument performance and data quality. The metabolites were separated on Amide XBridge HPLC column (100 mm×4.6 mm, 3.5 μm, Waters, USA). The mobile phase consisted of solvent A (water: acetonitrile=95:5, 5 mM ammonium acetate, pH=9) and solvent B (acetonitrile). The gradient elution program is as follows: 0-3 min, 85% B; 3-6 min, 85%-30% B; 6-15 min, 30%-2% B; 15-18 min, 2% B; 18-19 min, 2-85% B; 19-30 min 85% B. The negative ESI mode was performed, and a m/z range of 50 to 1,000 Da was used for the product ion scan.

To identify the metabolites, the acquired accurate precursor (mass tolerance < 10 ppm), retention period, and fragment ions (mass tolerance < 20 ppm) should match with the standard metabolome databases, such as the Human Metabolome Database, MassBank, and METLIN metabolite database [1-3]. For the key metabolites, such as pantothenate, further identification was conducted by comparing the mass features and retention time with the reference standards (Sigma). Metabolite abundance was acquired by computing the area under the curve for the quantification ion of the metabolites with MultiQuant 3.0. The quantitative data were normalized by the internal standard and tissue weights simultaneously. Multivariate statistical analysis was performed using SIMCA-P 13 software (Umetrics, Umeå, Sweden) with false discovery rate (FDR) correction applied to all p-values (Benjamini-Hochberg procedure). All the raw data and analysis have been supplied in Supplementary Material S2.

**Transepithelial electrical resistance (TEER) measurement**

4×10^4^ of Caco-2 cells were seeded into the upper chamber of 0.4 µm, 24-well transwell plates (Corning, USA). Culture medium was changed every other day up to 21 days. TEER was measured using Millicell^®^ ERS 3.0 Digital Voltohmmeter (Millipore, USA).

**FITC-dextran permeability measurement**

Mice were fasted for 4 h and then given FITC-dextran (FD-4, 500 mg/kg, Sigma-Aldrich, USA). 4 h after the administration, mice were anaesthetized, and blood samples were acquired. The serum FD-4 concentration was converted from the intensity of FITC which was measured by microplate spectrophotometer (excitation wavelength 480 nm; emission wavelength 520 nm, BioTek Instruments, USA). For cells, 200 µL FD-4 (1 mg/mL) was added into upper chamber and 600 µL HBSS was added into lower chamber of transwell, respectively. After 2 h of incubation at 37°C, 200 µL solution from lower chamber was collected for FD-4 measurement.

**Transmission electron microscopy**

Samples were fixed with 2.5% glutaraldehyde (Servicebio, China) for 2 hours at 4°C, followed by post-fixation in 1% OsO_4_ for 1 hour. After dehydration through a graded ethanol series, the samples were embedded in epoxy resin and polymerized at 60°C for 48 hours. Ultrathin sections (60-80 nm) were cut using an ultramicrotome and mounted on copper grids. The sections were stained with uranyl acetate and lead citrate to enhance contrast. Images were acquired using a transmission electron microscope HT7800 (Hitachi, Japan), operated at 80 kV. High-resolution micrographs were captured to analyze ultrastructural details, and image processing was performed using ImageJ for measurements of microvilli length and gap of AJC.

**Bioinformatic analyses**

Obtain expression matrices from GEO using GEOquery package in R. Filter low-expressed genes and convert probes to gene symbols via GPL annotation files, collapsing multi-probe genes by maximum expression. Visualize target gene expression using boxplots (ggplot2) stratified by experimental conditions.

**High-throughput virtual screening**

A virtual high-throughput screening was performed using an in-house natural product library to identify potential agonists of PANK3. The crystal structure of PANK3 (PDB: 6B3V) was prepared using the Protein Preparation Wizard within the Schrödinger Maestro suite, which included the assignment of bond orders, addition of hydrogens, and optimization of hydrogen-bonding networks. The Receptor Grid Generation module was used to define the active site for docking. A hierarchical screening strategy was employed to identify potential agonists. Initially, the entire library was docked using the Glide High-Throughput Virtual Screening (HTVS) mode. The resulting poses were ranked by the GlideScore, and the top 5% of compounds were advanced to a more rigorous docking step using the Glide Extra Precision (XP) mode. The final selection of top-ranking hits from the XP stage was further prioritized through visual inspection of their interaction patterns with key active site residues using PyMOL Molecular Graphics System (Version 3.5, Schrödinger), focusing on interactions indicative of agonist binding.

**Supplemental Tables**

**Supplemental Table S1** Antibodies used in this study

| **Protein** | **Source** | **Usage and dilution** |
| --- | --- | --- |
| GAPDH | Abcam ab181602 | 1:10000(WB) |
| Lamin B1 | Proteintech 66095-1-Ig | 1:10000(WB) |
| PANK1 | Proteintech 11768-1-AP | 1:1000(WB) |
| PANK2 | Origene TA501355 | 1:1000(WB) |
| PANK3 | Proteintech 12466-1-AP | 1:500(WB) 1:100(IHC/IF) |
| Occludin | Proteintech 27260-1-AP | 1:2000(WB) |
| ZO-1 | Invitrogen 61-7300 | 1:1000(WB) 1:100(IF) |
| E-cadherin | Cell Signaling Tech 3195 | 1:1000(WB) 1:200(IF) |
| β-catenin | Cell Signaling Tech 8480 | 1:1000(WB) |
| Claudin-1 | Proteintech 13050-1-AP | 1:1000(WB) 1:200(IF) |
| Claudin-4 | Abcam ab15104 | 1:1000(WB) |
| FLAG | Proteintech 20543-1-AP | 1:10000(WB) |
| c-Myc | Abcam ab32072 | 1:1000(WB) 1:200(IHC/IF) 2µg(ChIP) |
| Vimentin | Cell Signaling Tech 5741 | 1:1000(WB) 1:200(IF) |
| MMP9 | Proteintech 10375-2-AP | 1:1000(WB) |
| MMP8 | Abcam ab81286 | 1:1000(WB) |
| MMP2 | Proteintech 10373-2-AP | 1:1000(WB) |
| N-cadherin | Cell Signaling Tech 13116 | 1:1000(WB) 1:100(IF) |
| Fibronectin | Abcam ab2413 | 1:100(IF) |
| Twist | Abcam ab50887 | 1:500(WB) |
| Snail | Cell Signaling Tech 3879 | 1:1000(WB) |
| PI3K | Zenbio R22768 | 1:1000(WB) |
| Phos-PI3K | Cell Signaling Tech 4228 | 1:1000(WB) |
| AKT | Proteintech 10176-2-AP | 1:1000(WB) |
| Phos-AKT | Cell Signaling Tech 4060 | 1:1000(WB) |
| ERK1/2 | Cell Signaling Tech 4695 | 1:1000(WB) |
| Phos-ERK1/2 | Cell Signaling Tech 4370 | 1:1000(WB) |
| Goat-anti rabbit IgG, Alexa Fluro 594 | Invitrogen A-11012 | 1:500(IF) |
| Goat-anti rabbit IgG, Alexa Fluro 555 | Invitrogen A-31572 | 1:500(IF) |
| Goat-anti rabbit IgG HRP | Bioworlde BS13278 | 1:10000(WB) |
| Goat-anti mouse IgG HRP | Bioworlde BS13478 | 1:10000(WB) |

**Supplemental Table S2** Primer sequences used for qPCR

| **Gene** | **Forward primer (5’-3’)** | **Reverse primer (5’-3’)** |
| --- | --- | --- |
| **Human** | | |
| *PANK3* | TTTTGGCCGAAGAGGGAACTT | TAGCACCGTCTGCAATGTTGA |
| *GAPDH* | GGAGCGAGATCCCTCCAAAAT | GGCTGTTGTCATACTTCTCATGG |
| **Mouse** | | |
| *PANK1* | GTTCGCCCAGCATGATTCTC | CTTAACCAGGGTTCCACCGAT |
| *PANK2* | GAGGCGGAGAGTGTGAGAC | CCAAGGTTCCTCCAATATCCAAG |
| *PANK3* | GGACATTGGAGGAACGCTAGT | ACATCCCGAATGCCAGTAGAT |
| *MMP9* | CGTCGTGATCCCCACTTACT | AACACACAGGGTTTGCCTTC |
| *MMP8* | TCTTCCTCCACACACAGCTTG | CTGCAACCATCGTGGCATTC |
| *IL-1β* | GCAACTGTTCCTGAACTCAACT | ATCTTTTGGGGTCCGTCAACT |
| *IL-11* | TGTTCTCCTAACCCGATCCCT | CAGGAAGCTGCAAAGATCCCA |
| *Spp1* | AGCAAGAAACTCTTCCAAGCAA | GTGAGATTCGTCAGATTCATCCG |
| *Twist2* | CGCTACAGCAAGAAATCGAGC | GCTGAGCTTGTCAGAGGGG |
| *Snai1* | CACACGCTGCCTTGTGTCT | GGTCAGCAAAAGCACGGTT |
| *Fn1* | ATGTGGACCCCTCCTGATAGT | GCCCAGTGATTTCAGCAAAGG |
| *Tgf-β* | CTCCCGTGGCTTCTAGTGC | GCCTTAGTTTGGACAGGATCTG |
| *Pdgfra* | AGAGTTACACGTTTGAGCTGTC | GTCCCTCCACGGTACTCCT |
| *GAPDH* | GTGGCAAAGTGGAGATTGTTGCC | GATGATGACCCGTTTGGCTCC |
| **ChIP-PCR** | | |
| *F1* | TTAACTCCTCAGACCGTGCC | GGAACGCGTTATGTTCATGGG |
| *F2* | ATGCATCTCGGGCTTTGTAGT | GGGGATTGTAGTTCTCCGGTT |
| *F3* | ACGAACCGGAGAACTACA | CGCCGCACTGAGAAATAG |

**References**

[1]	WANG Q, WU M, LI H, RAO X, AO L, WANG H, et al. Therapeutic targeting of glutamate dehydrogenase 1 that links metabolic reprogramming and Snail-mediated epithelial-mesenchymal transition in drug-resistant lung cancer[J]. Pharmacol Res, 2022, 185: 106490.<https://doi.org/10.1016/j.phrs.2022.106490>

[2]	XU C, LIAO M, ZHANG S, CHEN Y, SHULAI X, WANG G, et al. The Comorbidity of Depression and Diabetes Is Involved in the Decidual Protein Induced by Progesterone 1 (Depp1) Dysfunction in the Medial Prefrontal Cortex[J]. Metabolites, 2025, 15(1).<https://doi.org/10.3390/metabo15010034>

[3]	ZHANG Y, LU W, WANG Z, ZHANG R, XIE Y, GUO S, et al. Reduced Neuronal cAMP in the Nucleus Accumbens Damages Blood-Brain Barrier Integrity and Promotes Stress Vulnerability[J]. Biol Psychiatry, 2020, 87(6): 526-537.<https://doi.org/10.1016/j.biopsych.2019.09.027>
